# Synergistic Impacts of Phosphorus Deficiency Coupled with Thermal and High-Light Stress on Physiological Profiles of Cultivated *Saccharina japonica*

**DOI:** 10.3390/plants14101412

**Published:** 2025-05-08

**Authors:** Jing Zhang, Xiaonan Wang, Xingyue Ren, Xu Gao, Jingyu Li

**Affiliations:** Key Laboratory of Mariculture, Ministry of Education, Fisheries College, Ocean University of China, Qingdao 266003, China; zhangjing2722@stu.ouc.edu.cn (J.Z.); wxn2516@stu.ouc.edu.cn (X.W.); renxingyue1998@163.com (X.R.)

**Keywords:** *Saccharina japonica*, phosphorus deficiency, thermal and high-light stress, growth, photosynthesis, phosphatase, metabolomics

## Abstract

Global kelp farming is garnering growing attention for its contributions to fishery yields, environmental remediation, and carbon neutrality efforts. Kelp farming systems face escalating pressures from compounded climatic and environmental stressors. A severe outbreak disaster caused extensive kelp mortality and significant economic losses in Rongcheng, China, one of the world’s largest kelp farming areas. This study investigated the growth and physiological responses of *Saccharina japonica* to combined stressors involving three levels of N:P ratios (10:1 as a control; 100:1 and 500:1 to represent phosphorus deficiency stress) and two temperature/light regimes (12 °C, 90 μmol photons m^−2^ s^−1^ as a control, and 17 °C, 340 μmol photons m^−2^ s^−1^ to represent thermal and high-light stress). The results demonstrated that phosphorus deficiency significantly inhibited the relative growth rate of kelp (24% decrease), and the strongest growth inhibition in kelp was observed at the N:P ratio of 500:1 combined with thermal and high-light stress. The algal tissue was whitened due to its progressive disintegration under escalating stress, coupled with damage to its chloroplasts and nucleus ultrastructures. Phosphorus-deficiency-induced declines in photochemistry (27–56% decrease) and chlorophyll content (63% decrease) were paradoxically and transiently reversed by thermal and high-light stress, but this “false recovery” accelerated subsequent metabolic collapse (a 60–75% decrease in the growth rate and a loss of thallus integrity). Alkaline phosphatase was preferentially activated to cope with phosphorus deficiency combined with photothermal stress, while acid phosphatase was subsequently induced to provide auxiliary support. *S. japonica* suppressed its metabolism but upregulated its nucleotides under phosphorus deficiency; however, the energy/amino acid/coenzyme pathways were activated and a broad spectrum of metabolites were upregulated under combined stressors, indicating that *S. japonica* employs a dual adaptive strategy where phosphorus scarcity triggers metabolic conservation. Thermal/light stress can override phosphorus limitations by activating specific compensatory pathways. The findings of this study provide a foundation for the sustainable development of kelp farming under climate and environmental changes.

## 1. Introduction

Climatic and environmental changes, coupled with anthropogenic activities, are increasingly disrupting marine ecosystems, with profound consequences for seaweed ecology and aquaculture [1]. Globally, seaweed populations face multiple stressors, including rising ocean temperatures, increased light intensity and UV radiation, ocean acidification, and altered nutrient dynamics, each exerting significant impacts [2]. For example, elevated sea temperatures induce thermal stress, impairing photosynthesis, nutrient uptake, and metabolic processes in seaweeds. This leads to reduced growth rates and heightened disease susceptibility [1]. Such temperature shifts also disrupt cellular homeostasis, exacerbating oxidative stress, especially when combined with intense light exposure, thereby threatening the economic viability of seaweed aquaculture [3,4]. Concurrently, enhanced ultraviolet (UV) radiation damages DNA, reduces photosynthetic efficiency, and triggers oxidative stress [5]. Ocean acidification, driven by rising atmospheric CO_2_, further compromises seaweed growth and photosynthetic capacity [6]. Meanwhile, coastal eutrophication, aggravated by nutrient runoff, promotes frequent harmful algal blooms, destabilizing seaweed cultivation environments [7]. Nutrient imbalances, particularly excess nitrogen relative to phosphorus (N:P ratio), also hinder the productivity of cultivated seaweeds [8]. Although phosphorus is required in smaller quantities compared to nitrogen, it is vital for energy transfer, photosynthesis, and the synthesis of nucleic acids and phospholipids [9]. Phosphorus deficiency severely limits growth and photosynthetic efficiency, as demonstrated by Zhou et al. [10], who observed significant declines in *Gracilariopsis lemaneiformis* under low-phosphorus conditions. These cumulative stressors endanger both natural seaweed habitats and aquaculture sustainability, where environmental stability is critical for productivity and quality. However, existing research predominantly examines individual stressors in isolation, despite the reality that seaweeds in natural and farmed settings face multiple concurrent pressures. The lack of comprehensive studies on interactive stressor effects, particularly nutrient imbalances combined with environmental shifts, hinders our ability to predict and mitigate impacts on seaweed growth and yield, and long-term aquaculture sustainability.

*Saccharina japonica* is an economically important kelp, particularly in East Asia. Accounting for approximately 70% of global farmed kelp (*S. japonica*) production, China’s kelp cultivation is predominantly concentrated in Shandong Province’s Rongcheng region (>50% of the national kelp output) and Fujian Province’s Ningde area, where optimal temperature and nutrient conditions sustain high kelp yields [11]. It serves as a valuable source of essential nutrients iodine, calcium, and vitamins [12,13]. Beyond its nutritional value, *S. japonica* contributes to various industries, including pharmaceuticals, cosmetics, and biofuel production, with alginate extracted from it being a crucial component in many commercial products [14]. *S. japonica* also plays a vital ecological role in sequestering carbon, mitigating coastal erosion, and providing habitats for marine species [15]. However, the sustainability of *S. japonica* aquaculture is increasingly threatened by environmental changes, particularly those linked to climate change, such as rising sea temperatures, shifts in nutrient availability, and increased light intensity [16,17]. In recent years, seaweed aquaculture in Rongcheng, Shandong Province, has faced significant yield fluctuations, culminating in an unprecedented large-scale *S. japonica* farming disaster in 2021 [18]. Preliminary investigations suggest that a combination of rising sea temperatures, increased water transparency, and extremely low levels of phosphorus caused by harmful algal blooms accounted for the huge loss of cultivated kelp in Rongcheng [19]. Among these factors, an imbalance in the N:P ratio, particularly phosphorus deficiency, has been identified as a major factor. According to the investigation report on cultivated kelp disasters from the National Modern Agricultural Industry Algae Technology System of the Ministry of Agriculture of China, the N:P ratio was above 100:1 in most of the disaster-stricken areas, and even reached 300–500:1 in some specific areas (unpublished data). Despite this, there is a still limited understanding of how these environmental factors interact to cause large-scale mortality in *S. japonica*, especially regarding the interactive effects of nutrient imbalances and other environmental stressors. This knowledge gap limits our ability to fully grasp the adaptive capacities of *S. japonica* in the face of climate change.

To bridge this critical knowledge gap, we present a mechanistic hypothesis explaining the kelp aquaculture collapses in Rongcheng, Shandong: while phosphorus limitation constitutes the primary stressor, its synergistic interaction with elevated temperature and increased light intensity induces catastrophic physiological dysfunction. To validate this hypothesis, we conducted a series of controlled culture experiments with *S. japonica* under laboratory conditions, simulating the environmental parameters observed during the disaster period. By monitoring the growth, ultrastructure, pigment content, photosynthetic efficiency, related enzyme activities, and differential metabolites of *S. japonica*, this study aims to explore how these environmental stressors affect the growth and health of *S. japonica*. The findings will provide scientific insights into improving stress resistance in seaweed and offer potential strategies to tackle practical challenges in aquaculture.

## 2. Results

### 2.1. The Growth of S. japonica

**Figure 1:**
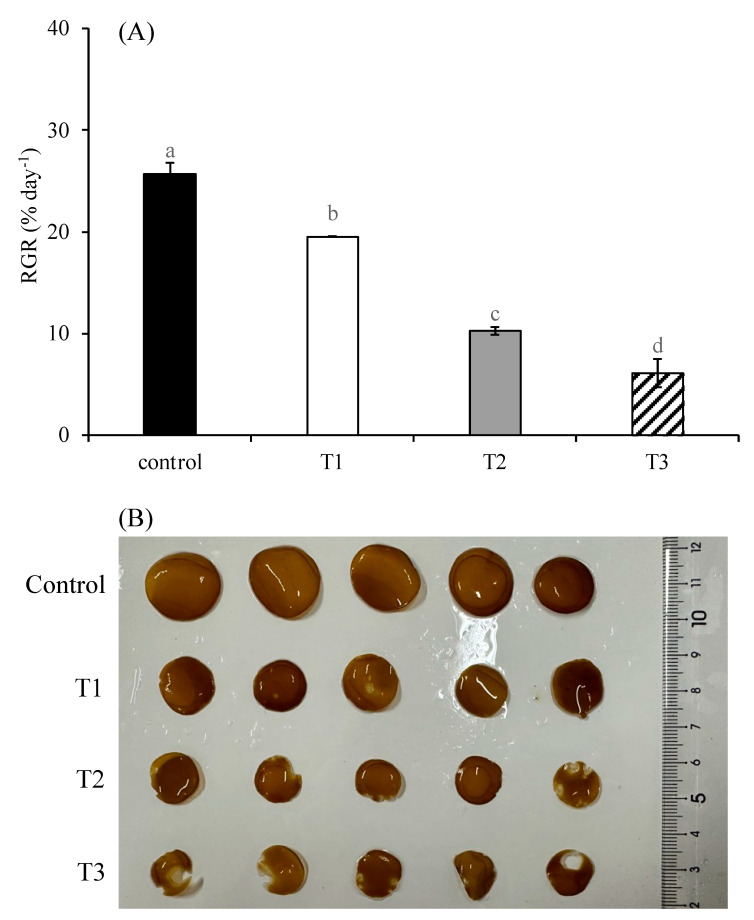
Relative growth rate (RGR) (**A**) and morphological status (**B**) of *S. japonica* after 6-day culture under four experimental conditions. Control indicates treatment under N:P 10:1, 12 °C, and 90 μmol photons m^−2^ s^−1^; T1 indicates treatment under N:P 100:1, 12 °C, and 90 μmol photons m^−2^ s^−1^; T2 indicates treatment under N:P 100:1, 17 °C, and 340 μmol photons m^−2^ s^−1^; T3 indicates treatment under N:P 500:1, 17 °C, and 340 μmol photons m^−2^ s^−1^. Data are presented as mean ± SE (n = 3). Different letters indicate significant differences among treatments at *p* < 0.05.

The relative growth rate (RGR) of *S. japonica*, shown in Figure 1A, was significantly inhibited by the elevated N:P ratio, as well as its synergistic effect with high temperature and high light intensity (*p* < 0.05). Notably, the growth-inhibitory effect became more pronounced with an increase in the N:P ratio, temperature, and light intensity. As the N:P ratio increased from 10:1 to 100:1, the RGR declined from 25.72% day^−1^ to 19.51% day^−1^ (*p* < 0.05). When the elevated N:P ratio was combined with high temperature and high light intensity, the RGR further decreased to 10.24% day^−1^ (*p* < 0.05). Under the condition of a N:P ratio of 500:1 in combination with high temperature and light intensity, the RGR dropped to the lowest value of 6.10% day^−1^ (*p* < 0.05).

The comparative morphological analysis of algal disks among treatments after cultivation is presented in Figure 1B. Compared to the control group, the algal disks in the T1 group became smaller, with some developing light-colored spots. The T2 group exhibited further reduced algal disks, with light-colored spots appearing along the margins and showing partial tissue disintegration. The T3 group displayed the smallest algal disks, with nearly all algal disks exhibiting marginal tissue disintegration.

**Figure 2:**
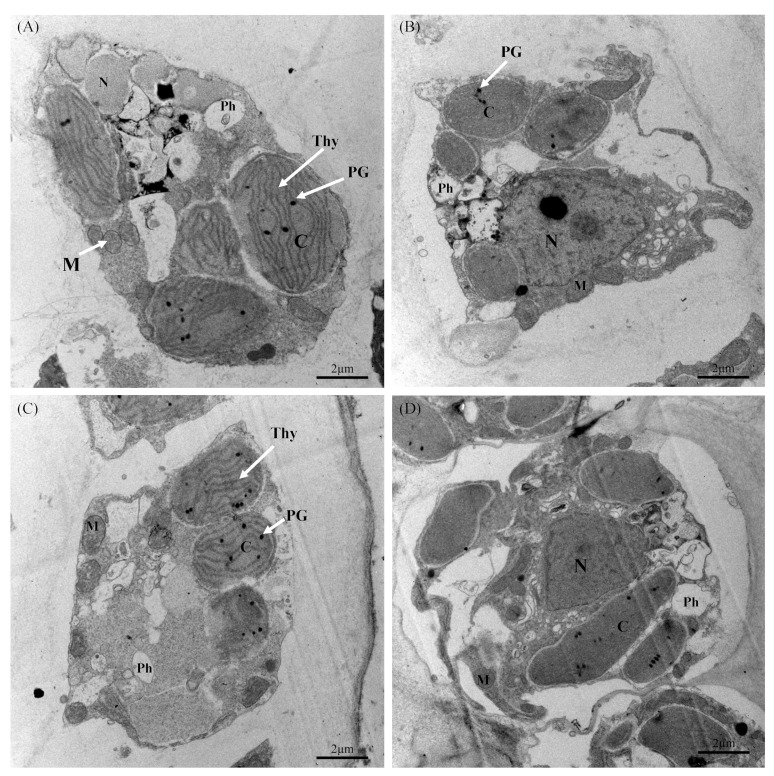
Ultrastructure of *S. japonica* cells after 6-day culture under four experimental conditions. Photographs of control with T1, T2, and T3 are labeled (**A**–**D**). Symbols used correspond to those defined in Figure 1. N: nucleus; M: mitochondrion; C: chloroplast; PG: plastoglobulus; Thy: thylakoid; Ph: physode. Scale bar = 2 μm.

The ultrastructural analysis of cells under various treatments is shown in Figure 2. In the control group, the cells displayed a typical ultrastructure, with intact cell walls, nuclei, mitochondria, and physodes (Figure 2A). Chloroplasts were well preserved, with organized thylakoids and visible plastoglobuli. Although the overall cellular architecture of the T1 group remained comparable to that of the control group, minor structural changes were observed (Figure 2B). Notably, both the nucleus and nucleolus appeared enlarged, and the chloroplasts showed a slight reduction in size compared to the control group. The cells in the T2 group exhibited more pronounced structural alterations, including disrupted chloroplasts and mitochondria, the complete absence of a nucleus, and a reduction in physode size relative to the control group (Figure 2C). In the T3 group, the cells underwent severe structural damage, characterized by highly disrupted chloroplasts and nucleoli disappearing (Figure 2D).

### 2.2. Pigment Content of S. japonica

**Figure 3:**
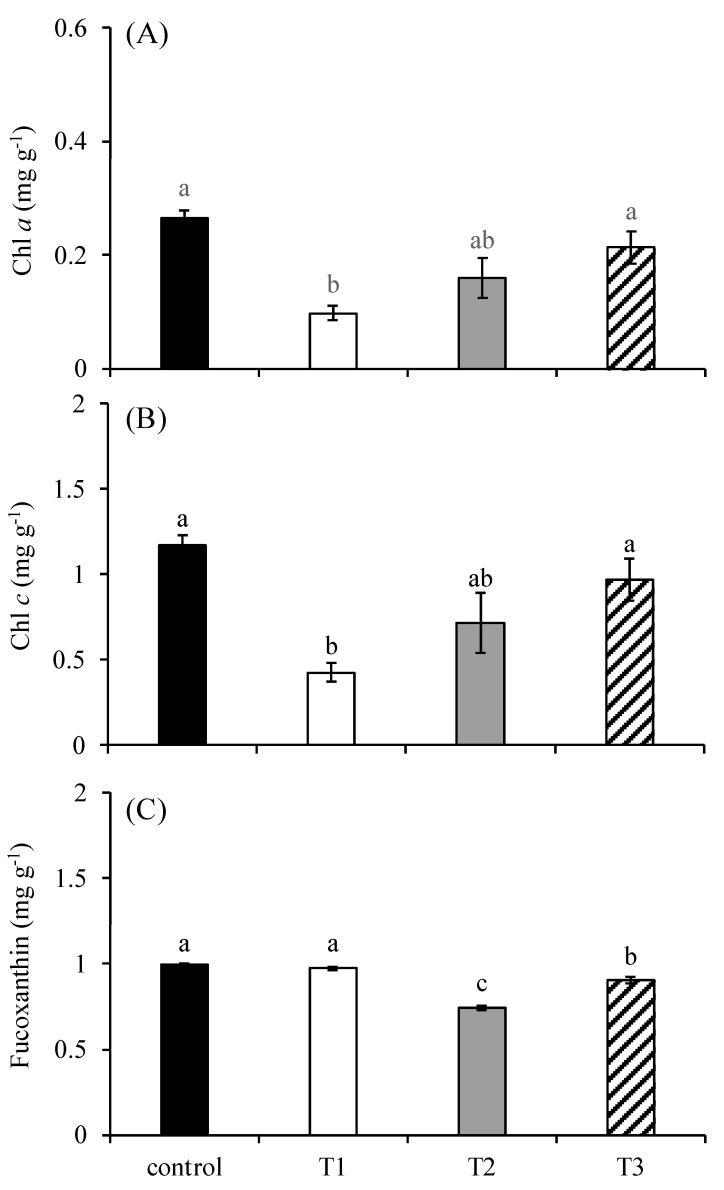
The content of chlorophyll *a* (**A**), chlorophyll *c* (**B**), and fucoxanthin (**C**) in *S. japonica* after a 6-day culture under four experimental conditions. The symbols used correspond to those defined in Figure 1. The data are presented as the mean ± SE (n = 3). Different letters indicate significant differences among treatments at *p* < 0.05.

There were significant differences in the contents of chlorophyll *a* (Figure 3A), chlorophyll *c* (Figure 3B), and fucoxanthin (Figure 3C) among treatments (*p* < 0.05). The contents of chlorophyll *a* and chlorophyll *c* in the control group were 0.26 mg g^−1^ and 1.17 mg g^−1^, respectively. Their contents in the T1 group were 0.10 mg g^−1^ and 0.43 mg g^−1^, respectively, which were significantly lower than those in the control group and the T3 group. Their contents in the T2 group were 0.16 mg g^−1^ and 0.72 mg g^−1^, respectively, which were only significantly lower than those in the control group (*p* < 0.05). Their contents in the T3 group were 0.21 mg g^−1^ and 0.97 mg g^−1^, respectively, which were only significantly higher than those in the T1 group (*p* < 0.05). The content of fucoxanthin was 0.74 mg g^−1^ in the T2 group, which was significantly lower than that of any other treatments. It was 0.91 mg g^−1^ in the T3 group, which was higher than that in the T1 group but significantly lower than those of in the control group and the T1 group (*p* < 0.05).

### 2.3. Fluorescence Parameters of Chlorophyll

**Figure 4:**
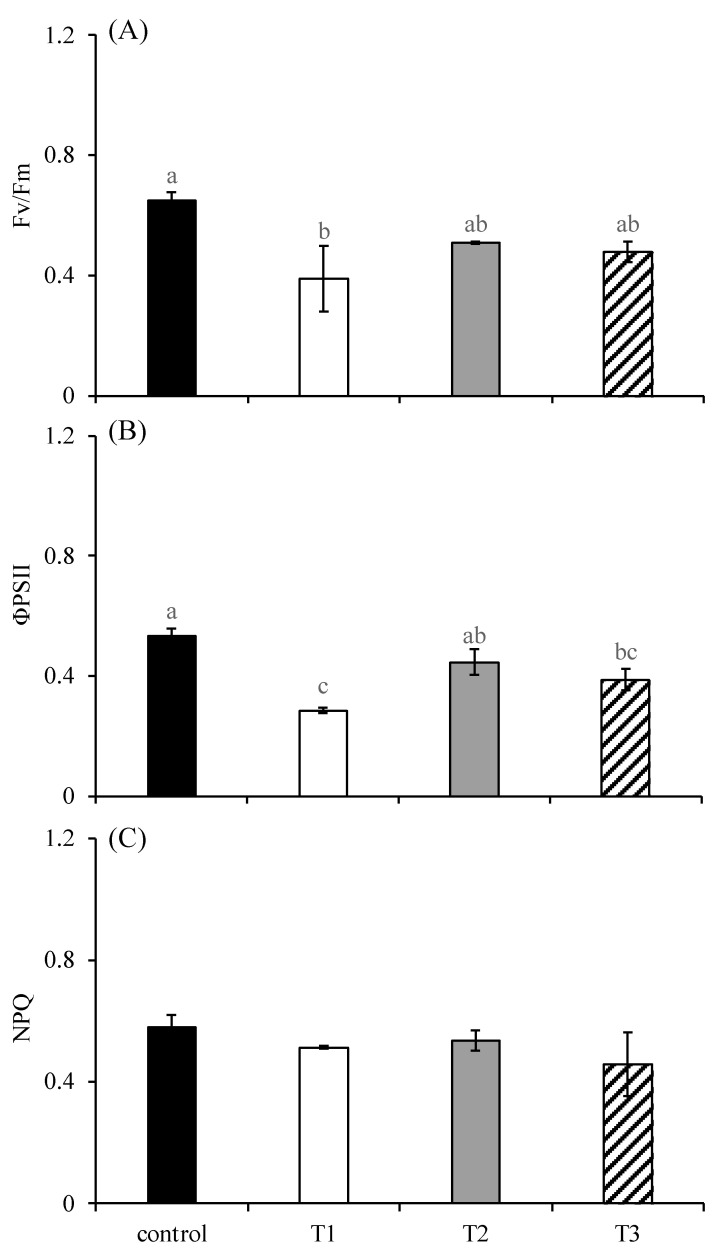
Chlorophyll fluorescence parameters (Fv/Fm (**A**); ΦPSII (**B**); NPQ (**C**)) of *S. japonica* after a 24 h culture under four experimental conditions. The symbols used correspond to those defined in Figure 1. The data are presented as the mean ± SE (n = 3). Different letters indicate significant differences among treatments at *p* < 0.05.

The F_v_/F_m_ of *S. japonica* differed significantly among treatments (*p* < 0.05; Figure 4A). The F_v_/F_m_ of the T1 group was significantly lower than that of the control group. However, there was no significant difference in the F_v_/F_m_ among the control, T2, and T3 groups. Additionally, the F_v_/F_m_ among the T1, T2, and T3 groups has no significant differences. The ΦPSII of *S. japonica* differed significantly among treatments (*p* < 0.05; Figure 4B). The ΦPSII of the T1 group was significantly lower than those of the control group and T3 group (*p* < 0.05). The ΦPSII of the T3 group was significantly lower than that of the control group (*p* < 0.05). There was no significant difference in ΦPSII among other treatments (*p* > 0.05). The NPQ of *S. japonica* showed no significant difference among treatments (*p* > 0.05; Figure 4C).

### 2.4. Phosphatase Activity

**Figure 5:**
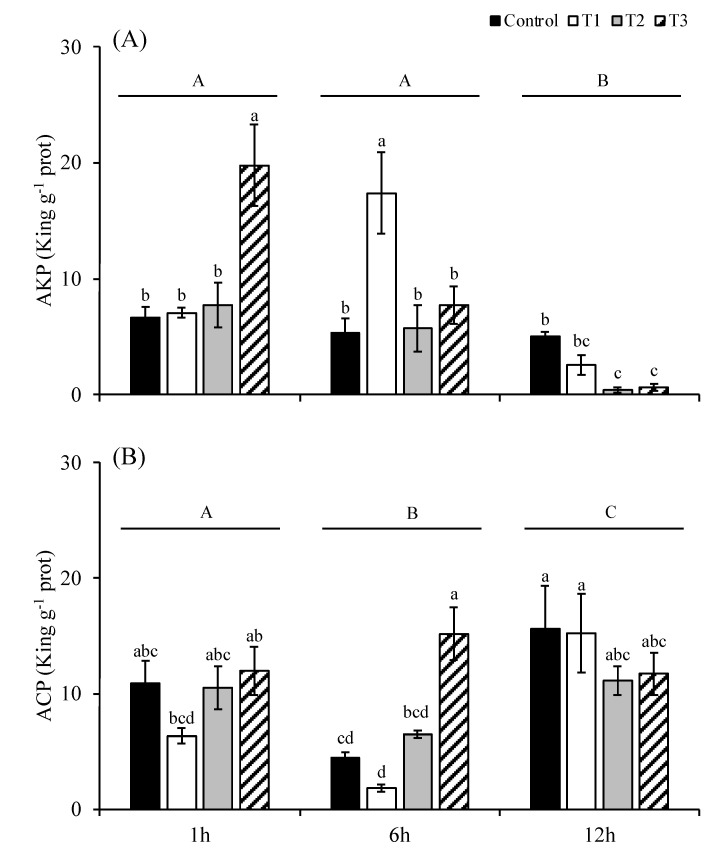
The activities of alkaline phosphatase (**A**) and acid phosphatase (**B**) of *S. japonica* after 1, 6, and 12 h of culture under four experimental conditions. The symbols used correspond to those defined in Figure 1. The data are presented as the mean ± SE (n = 3). Different letters indicate significant differences among treatments at *p* < 0.05. Different capital letters indicate statistical significance (*p* < 0.05) among treatments at 1 h, 6 h and 12 h.

The alkaline phosphatase (AKP) activity of *S. japonica* showed significant differences at the 1st hour, 6th hour and 12th hour (*p* < 0.05; Figure 5A). The AKP activities at the 1st and 6th hour were significantly higher than those at the 12th hour (*p* < 0.05). The difference in AKP activities between the 1st and the 6th hours was not significant (*p* > 0.05). The AKP activities in the T3 group at the 1st hour (19.78 king unit g^−1^ prot) and in the T1 group at the 6th hour (17.39 king unit g^−1^ prot) were significantly higher than those of the other treatments (*p* < 0.05). The AKP activities in the T2 and T3 groups at the 12th hour were significantly lower than those of the control group.

The acid phosphatase (ACP) activity of *S. japonica* was significantly different at the 1st hour, 6th hour, and 12th hour (*p* < 0.05; Figure 5B). The ACP activities at the 12th hour were significantly higher than those at the 1st and the 6th hours. The ACP activities at the 6th hour were significantly higher than those at the 1st hour. Among the treatments at each time, there was no significant difference in the ACP activities among treatments at the 1st and the 6th hours (*p* > 0.05), except that the ACP activity of the T3 group at the 6th hour was significantly higher than that of any other treatments at the same time (*p* < 0.05).

### 2.5. Metabolomic Profiles

**Figure 6:**
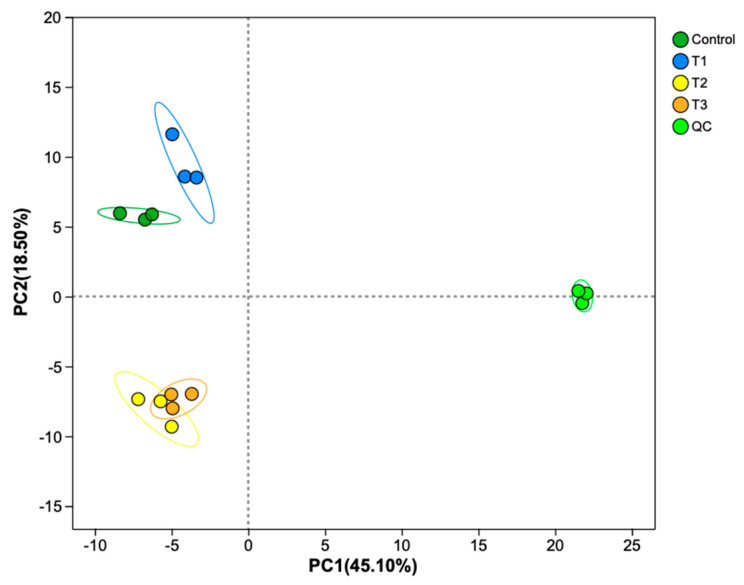
A principal component analysis (PCA) plot of *S. japonica* metabolites after a 6-day culture under four experimental conditions. The symbols used correspond to those defined in Figure 1.

**Figure 7:**
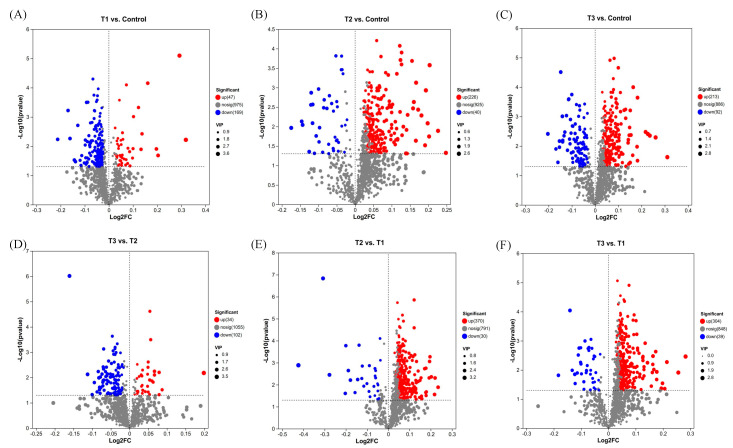
An expression volcano map of upregulated and downregulated differential metabolites in the comparisons between the T1 and control groups (**A**), between the T2 and control groups (**B**), between the T3 and control groups (**C**), between the T3 and T2 groups (**D**), between the T2 and T1 groups (**E**), and between the T3 and T1 groups (**F**). The symbols used correspond to those defined in Figure 1. Blue dots represent downregulated metabolites, red dots represent upregulated metabolites, and gray dots represent metabolites with unchanged expression levels.

Principal component analysis (PCA) revealed changes in metabolites among the control, T1, T2, and T3 groups. PC1 accounted for 45.10% of the explained variance, exhibiting a modest association with basal nutrient availability. In contrast, PC2 (18.5% of variance explained) distinctly captured the metabolic signatures of high-temperature/high-light stress, with its lower variance contribution offset by heightened biological relevance (Figure 6). PCA revealed distinct clustering patterns among treatment groups, with triplicate biological replicates consistently forming tight clusters, demonstrating minimal intragroup variability. Specifically, the T2 and T3 groups exhibited close spatial proximity in the PCA score plot, while both the control and T1 groups remained distinctly separated from the other experimental groups. This spatial distribution pattern suggests minor metabolic profile differences between the T2 and T3 groups, but substantial compositional and quantitative variations in metabolites when comparing the control and T1 groups to the other treatments. In total, 1259 metabolites were identified. Specifically, there were 1191 differential metabolites (DMs): 47 were upregulated and 169 were downregulated between the T1 and control groups (Figure 7A); 226 were upregulated and 40 were downregulated between the T2 and control groups (Figure 7B); 213 were upregulated and 92 were downregulated between the T3 and control groups (Figure 7C); 34 were upregulated and 102 were downregulated between the T3 and T2 groups (Figure 7D); 370 were upregulated and 30 were downregulated between the T2 and T1 groups (Figure 7E); and 304 were upregulated and 39 were downregulated between the T3 and T1 groups (Figure 7F). These results show a trend whereby, in the groups with high-temperature and high-light treatment (T2 vs. control, T3 vs. control, and T2 vs. T1), the upregulated metabolites are greater in number than the downregulated metabolites, while in the groups with only an increase in the N:P ratio (T1 vs. control and T3 vs. T2), the downregulated metabolites are greater in number than the upregulated metabolites.

**Figure 8:**
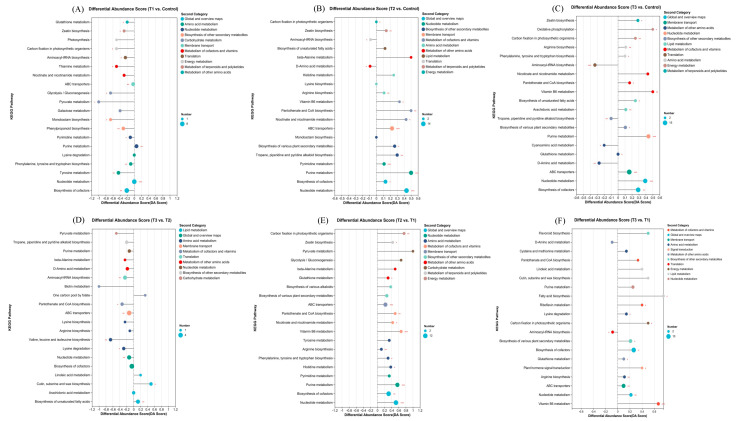
Differential abundance score plots of KEGG pathways in the comparisons between the T1 and control groups (**A**), between the T2 and control groups (**B**), between the T3 and control groups (**C**), between the T3 and T2 groups (**D**), between the T2 and T1 groups (**E**), and between the T3 and T1 groups (**F**). The symbols used correspond to those defined in Figure 1. An asterisk (*), two asterisks (**) and three asterisks (***) indicated a significance level of *p* < 0.05, *p* < 0.01 and *p* < 0.001, respectively.

The DMs were subjected to enrichment analysis by searching the KEGG pathway database (Figure 8). The DMs were enriched in 12 KEGG pathways at the second-category level, including global and overview maps, amino acid metabolism, nucleotide metabolism, the biosynthesis of other secondary metabolites, carbohydrate metabolism, membrane transport, the metabolism of cofactors and vitamins, translation, energy metabolism, the metabolism of terpenoids and polyketides, the metabolism of other amino acids, and lipid metabolism. Six of these pathways, including global and overview maps, amino acid metabolism, nucleotide metabolism, the biosynthesis of other secondary metabolites, membrane transport, and the metabolism of cofactors and vitamins, were enriched across almost all group comparisons. Energy metabolism and the metabolism of terpenoids and polyketides were enriched in the comparisons between T1 and the control, T2 and the control, T3 and the control, and T2 and T1. Translation was enriched in the comparisons between T1 and the control, T2 and the control, T3 and the control, T3 and T2, and T3 and T1. The metabolism of other amino acids and lipid metabolism were enriched in the comparisons between T1 and the control, T2 and the control, T2 and T1, T3 and T2, and T3 and T1. Carbohydrate metabolism was enriched in the comparisons between T1 and the control, T2 and T1, and T3 and T2. Lipid metabolism was enriched in the comparisons between T2 and the control, T3 and the control, T3 and T2, and T3 and T1. In the comparisons between T1 and the control and T3 and T2, most metabolites in these KEGG pathways were more downregulated. In the comparisons between T2 and the control, T3 and the control, T2 and T1, and T3 and T1, most metabolites in these KEGG pathways were more upregulated.

**Figure 9:**
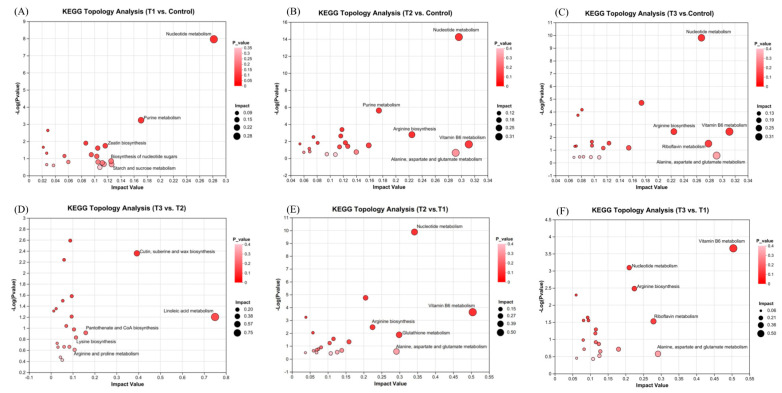
Topological analysis of KEGG pathways in the comparisons between the T1 and control groups (**A**), between the T2 and control groups (**B**), between the T3 and control groups (**C**), between the T3 and T2 groups (**D**), between the T2 and T1 groups (**E**), and between the T3 and T1 groups (**F**). The symbols used correspond to those defined in Figure 1.

The KEGG topology analysis of differential metabolites identified 20 significant pathways for each group, with the top five most important pathways highlighted in Figure 9. Among these pathways, the nucleotide metabolism pathways emerged as the most critical across all comparisons between the three treatment groups (T1, T2, T3) and the control group, as well as when both the T2 and T3 groups were compared to the T1 group. Additionally, the amino acid metabolism pathways (arginine biosynthesis and alanine, aspartate, and glutamate metabolism), along with the vitamin B6 metabolism pathway, were identified as key pathways in both the T2 and T3 groups compared to the control group. The amino acid metabolism pathways and the vitamin B6 metabolism pathway were also prominent when comparing both T2 and T3 to T1. However, the pathways of cutin, suberin and wax biosynthesis, linoleic acid metabolism, pantothenate and CoA biosynthesis, lysine biosynthesis, and arginine and proline metabolism were observed only in the comparison between T3 and T2, which differed from the pathways observed in the other comparisons. These findings suggest that, under high-temperature and high-light conditions, the metabolic responses of *S. japonica* to phosphorus deficiency differ from those observed under normal-temperature and -light conditions.

## 3. Discussion

### 3.1. Effects of Phosphorus Deficiency on the Growth and Physiological Responses of Cultivated S. japonica

This study systematically investigates the impacts of phosphorus deficiency and its synergistic interactions with elevated temperature and high light intensity on the growth and physiological responses of cultivated *S. japonica*. The experimental results demonstrated a statistically significant reduction (*p* < 0.05) in the relative growth rate (RGR) of *S. japonica* across all phosphorus-deficient treatments (T1–T3) compared to the control groups. Notably, this growth inhibition was markedly exacerbated when phosphorus deficiency co-occurred with increased temperature and high light intensity (T2 and T3 treatments), revealing important stressor interactions. As a fundamental structural component of nucleic acids and ATP, phosphorus plays critical roles in various metabolic processes of marine macroalgae [20]. Our findings align with previous studies showing that phosphorus limitation typically manifests through reduced growth rates, diminished thallus size, and morphological abnormalities in kelp species [21]. The observed phenotypic responses in *S. japonica* under phosphorus-deficient conditions, including stunted growth and blade deformities, exhibited striking similarities to the morphological symptoms documented during the 2021 kelp disaster along the Rongcheng coast. This morphological congruence provides compelling evidence that phosphorus deficiency may serve as a primary driver of the observed kelp disaster. The synergistic effects of concurrent environmental stressors (temperature and light) likely amplified the physiological impacts, suggesting that multi-stressor interactions should be considered in understanding large-scale declines in the kelp population.

Phospholipids are fundamental structural components of thylakoid membranes, playing a critical role in maintaining membrane integrity and facilitating efficient electron transport within photosystem II (PSII) and photosystem I (PSI) [22]. The ultrastructural analysis revealed that chloroplasts were the most severely affected organelles under phosphorus deficiency, suggesting that phosphorus limitation primarily impairs photosynthetic function in *S. japonica*. Previous studies have reported that phosphorus deficiency disrupts algal photosynthesis through pigment degradation (reducing light-harvesting capacity), electron transport chain impairment (limiting ATP/NADPH synthesis), and RuBP regeneration inhibition (constraining Calvin cycle activity) [23,24,25]. Our results are consistent with those of previous reports. Our quantitative analysis revealed a 63–64% reduction in the chlorophyll contents in T1 (*p* < 0.01) and a 17–19% decline in T2 and T3 (though not statistically significant), and a 9–26% loss of fucoxanthin content in T2 and T3 (*p* < 0.05) was observed compared to the controls. These pigment dynamics are characteristic of phosphorus-starved seaweeds and are strongly correlated with diminished photosynthetic efficiency [25,26]. Collectively, our results establish phosphorus deficiency as a key regulator of kelp photopigment metabolism, with implications for productivity in nutrient-limited marine environments.

Furthermore, our results demonstrate significant photochemical impairment under phosphorus-deficient conditions, as evidenced by the pronounced suppression of both the maximum quantum yield (F_v_/F_m_) and effective quantum yield of PSII (ΦPSII) in T1. Although less severe, similar declining trends were observed in T2 and T3 (albeit statistically non-significant), collectively indicating disrupted electron transport capacity under phosphorus-limiting conditions. These findings align with recent work on *G*. *lemaneiformis*, where phosphorus deficiency similarly reduced F_v_/F_m_ values, attributed to light-dependent ATP depletion and subsequent PSII dysfunction [27]. As a fundamental constituent of ATP and NADPH, the key energy carriers in photosynthesis [28], phosphorus scarcity directly curtails their synthesis. This mechanistic link explains the observed decline in photosynthetic efficiency and growth inhibition in phosphorus-starved macroalgae [29]. We therefore conclude that the reduced relative growth rate (RGR) in *S. japonica* primarily stems from phosphorus deficiency-induced photosynthetic impairment, mediated through electron transport chain disruption (quantified via F_v_/F_m_ and ΦPSII), energy molecule (ATP/NADPH) limitation, and consequent carbon fixation suppression. Collectively, the transient photosynthetic improvement (‘false recovery’) that manifested through higher Fv/Fm and ΦPSII ratios under T2/T3 conditions likely represents a stress-adaptive ATP investment in PSII repair pathways, analogous to *Ulva prolifera*’s response to copper toxicity [30]. This survival strategy, however, initiates a metabolic cascade: chronic ATP diversion progressively exhausts nucleotide reservoirs essential for genomic maintenance (Figure 9), culminating in irreversible cellular collapse. These findings substantiate the algal ‘metabolic gambling’ paradigm [31], where organisms evolutionarily optimize short-term stress mitigation at the expense of long-term viability through strategic resource reallocation.

As a photosynthetic macroalga, *S. japonica* requires both inorganic carbon (CO_2_/HCO_3_^−^) and critical macronutrients, particularly nitrogen and phosphorus, to support its primary metabolic processes, including photosynthesis, protein synthesis, and nucleic acid production. To elucidate the physiological response of *S. japonica* to phosphorus deficiency, we evaluated the temporal dynamics of alkaline phosphatase (AKP) and acid phosphatase (ACP) activities at 1, 6, and 12 h under varying environmental conditions. Our results revealed the distinct activation patterns between the two phosphatases, with AKP exhibiting greater sensitivity to phosphorus deficiency than ACP. Notably, the AKP activity peaked at 1 h under severe phosphorus deficiency combined with high temperature and light (the T3 group), where it was significantly elevated compared to that in the other groups (*p* < 0.05). This rapid induction suggests an immediate adaptive response to extracellular P scarcity, enabling the hydrolysis of organic phosphates to replenish cellular P pools [32]. In contrast, the ACP activity remained stable across most treatments during the initial hours, showing a delayed increase only after 6 h in the T3 group. This temporal disparity implies that ACP primarily facilitates intracellular phosphorus recycling when external phosphorus sources are depleted [33]. AKP serves as a first-line defense against acute P deficiency, rapidly scavenging extracellular phosphorus. ACP acts as a secondary response, sustaining phosphorus homeostasis through internal recycling during prolonged stress. The differential regulation of AKP and ACP underscores a sophisticated phosphorus acquisition strategy in *S. japonica*, where rapid extracellular phosphorus mobilization (AKP) is later complemented by intracellular recycling (ACP) to optimize phosphorus utilization under stress. In addition to the direct negative effects of phosphorus deficiency on kelp growth, our results demonstrate that phosphorus limitation also suppressed nitrogen uptake in *S. japonica* according to the Redfield ratio requirements, thereby inducing artificial nitrogen deficiency despite ambient nitrogen availability. This coupled nitrogen and phosphorus co-limitation inevitably leads to growth inhibition through two synergistic mechanisms: constraining biosynthetic capacity due to phosphorus shortage (affecting ATP, nucleic acids, etc.) and disrupting nitrogen assimilation (e.g., glutamine synthetase requires ATP).

Our untargeted metabolomic analysis revealed that phosphorus deficiency in *S. japonica* induces complex metabolic reprogramming characterized by the global downregulation of metabolites, consistent with the known phosphorus-limiting effects in algae [31,34], while the concurrent elevated-temperature and -light conditions trigger compensatory metabolic responses. Under phosphorus deficiency alone (T1), we observed widespread metabolic suppression, with the notable exception of upregulated nucleotide metabolism, indicating preferential phosphorus allocation to essential biomolecules. In contrast, phosphorus-deficient groups exposed to elevated temperature and high light intensity (T2/T3) showed the significant activation of multiple pathways, including energy metabolism (TCA cycle intermediates), amino acid biosynthesis, and coenzyme/vitamin metabolism, with T2 exhibiting near-universal metabolite upregulation relative to T1. These findings demonstrate that *S. japonica* employs a sophisticated adaptive strategy where phosphorus scarcity triggers metabolic conservation, but light/thermal stress can override these limitations by activating specific compensatory pathways [30,35], highlighting the organism’s ability to dynamically balance nutrient limitation and environmental stress through metabolic flexibility.

### 3.2. Synergistic Effect of Elevated Temperature and High Light Intensity with Phosphorusdeficiency on the Growth and Physiological Responses of Cultivated S. japonica

The concurrent fluctuations in the three cardinal determinants of the recruitment success of kelp, i.e., the thermal regime, photosynthetically active radiation, and nutrient stoichiometry, create complex stressor interactions that directly govern mariculture productivity. Through precisely controlled culture experiments replicating kelp disaster period conditions, we decoupled the individual and synergistic effects of these three key environmental drivers, identifying their hierarchical contributions to the observed kelp disaster.

Our findings demonstrate that the combined stressors of elevated temperature, high light intensity, and phosphorus deficiency synergistically reduced the relative growth rate of *S. japonica*, with particularly pronounced effects in the T2 and T3 treatment groups. This exacerbated growth inhibition appears to stem from two key physiological conflicts: (1) the metabolic acceleration–demand paradox and (2) oxidative stress accumulation. The metabolic paradox emerges when the thermal and photic stimulation of algal metabolism (evidenced by the increased chlorophyll content, enhanced F_v_/F_m_ and ΦPSII values, and widespread metabolite upregulation in the T2/T3 groups) creates heightened nutrient demands that cannot be met under phosphorus-limiting conditions [24,36]. This imbalance between metabolic activation and nutrient supply leads to disproportionate growth suppression. Previous studies have reported that a sufficient nitrogen and phosphorus supply can mitigate high-temperature damage to algal tissues by enhancing the antioxidant capacity of kelp (e.g., increasing SOD and CAT enzyme activities), thereby reducing oxidative stress induced by high-temperature stress [14,37]. Additionally, phosphorus may support kelp in maintaining its physiological activity under high temperatures by participating in energy metabolism (e.g., ATP synthesis) [38]. Based on these findings, it can be inferred that nutrient stress (e.g., nitrogen or phosphorus limitation) may be more lethal to kelp than temperature or light stress.

The thermal and photic stimulation simultaneously induced oxidative stress through elevated vitamin B6 metabolites (which serve as oxidative stress markers) [39] and caused ultrastructural damage to chloroplasts, the most affected organelle. The upregulation of coenzyme/vitamin pathways, particularly vitamin B6 metabolism, under combined stressors (T2/T3) suggests a critical role in mitigating oxidative damage. Vitamin B6 acts as a cofactor for antioxidant enzymes and stabilizes reactive oxygen species (ROS) scavengers [39]. For instance, in *U. prolifera*, vitamin B6 derivatives alleviate oxidative stress during high-temperature and copper exposure by enhancing glutathione synthesis [30]. Similarly, *S. japonica* may employ vitamin B6 to counteract ROS generated by photothermal stress, thereby delaying chloroplast degradation. This compensatory mechanism aligns with findings in diatoms, where vitamin B6 pathways are activated under phosphorus limitation to sustain redox homeostasis [31]. These findings align with the reported ROS accumulation mechanisms in *Kappaphycus alvarezii* under similar stress conditions [10]. These findings support a model where phosphorus deficiency primarily limits biosynthesis, while thermal/light stress both exacerbate this nutrient limitation through increased metabolic demand and cause additional oxidative damage. For example, in *Fucus serratus*, thermal stress induces metabolic acceleration but exacerbates nutrient demands, leading to growth suppression under nitrogen limitation [35]. Similarly, *G. lemaneiformis* exhibits reduced ATP synthesis under phosphorus deficiency, which synergizes with high-temperature stress to disrupt photosystem II repair mechanisms [27]. These studies highlight the universality of metabolic–nutrient conflicts in macroalgae under multi-stressor conditions. The chloroplast-targeted effects particularly highlight photosynthesis as the key vulnerability point under these combined stressors, ultimately leading to significantly greater growth inhibition than that caused by a single stressor. While the T1 group (phosphorus deficiency alone) showed no photodamage, the impaired ATP synthesis due to phosphorus limitation significantly reduced photosystem II (PSII) activity. Interestingly, contrary to conventional expectations, the combined stressors of high temperature and light intensity with phosphorus deficiency (T2) or extreme phosphorus deficiency (T3) did not produce statistically significant reductions in the photosynthetic capacity. This counterintuitive finding suggests that, under multiple stress conditions, the effects of thermal and light stress may mask the specific impacts of phosphorus limitation on photosynthetic function. Notably, while the chlorophyll content in the T2 and T3 groups was numerically lower than that in the controls, the lack of statistical significance implies the following: (i) phosphorus availability alone cannot fully explain the observed photosynthetic responses, and (ii) temperature and light intensity likely play compensatory or modifying roles in chlorophyll synthesis and maintenance. These complex interactions highlight the need for more comprehensive studies to elucidate the following: the threshold levels at which phosphorus limitation becomes the dominant stress factor, the potential protective mechanisms activated by thermal/light stress, and the metabolic trade-offs involved in acclimation to multiple stressors. The key trade-offs include (i) energy allocation to nucleotide synthesis (essential for DNA repair under stress) at the expense of carbohydrate storage, as observed in *Sargassum macrocarpum* [38], and (ii) the prioritization of antioxidant production (e.g., glutathione) over growth-related metabolites, a strategy documented in *Ulva compressa* under copper stress [40]. Future studies should focus on (i) identifying threshold levels of phosphorus limitation that trigger irreversible metabolic collapse, (ii) elucidating ROS signaling pathways linking thermal/light stress to chloroplast degradation, and (iii) exploring the epigenetic mechanisms underlying compensatory acclimation. For instance, transcriptomic analyses of *Ecklonia radiata* under combined stressors revealed heat shock proteins as critical mediators of cross-tolerance [37], a pathway warranting investigation in *S. japonica*.

### 3.3. Sustainable Kelp Aquaculture Under Climate and Environmental Changes: Challenges and Mitigation Strategies

Kelp aquaculture serves as a vital pillar of the blue economy, delivering essential services that include carbon sequestration, habitat restoration, and food security. However, the sector is increasingly threatened by the combined effects of climate change, such as ocean warming, acidification, and, more frequently, extreme weather events [41,42,43,44], and anthropogenic stressors, including eutrophication, heavy metal pollution, and microplastic contamination [40,45,46]. While the individual impacts of these stressors on kelp physiology are well documented, critical knowledge gaps remain regarding their synergistic effects on kelp. Specifically, three key areas require further investigation: (1) metabolic trade-offs under concurrent stressors, (2) physiological tolerance thresholds, and (3) capacity for compensatory acclimation. These gaps hinder our ability to predict and mitigate climate-driven declines in kelp aquaculture productivity.

Compounding these challenges, harmful algal blooms (HABs), driven by diverse taxa such as dinoflagellates, diatoms, and macroalgae, pose an underestimated yet growing threat to kelp farming systems worldwide. Regional HAB patterns exhibit distinct characteristics: in East Asia, recurrent blooms plague the Yellow Sea and the East China Sea [47]; in Southeast Asia, *Pyrodinium bahamense* outbreaks disrupt tropical waters [48]; in Europe, Dinophysis blooms affect the Baltic and North Seas [49]; in North America, *Karenia* brevis red tides and *Aureococcus anophagefferens* brevis brown tides occur [50,51], and in South America, *Alexandrium* blooms impact coastal aquaculture [52]. These events harm kelp through multiple mechanisms, including light attenuation, toxin release, oxygen depletion, and ecological shifts favoring competing species. A stark example occurred in Rongcheng, China, where a 2021 red tide event caused drastic shifts in N:P ratios, increased light penetration, and led to the extensive loss of kelp biomass, demonstrating the severe consequences of HABs when combined with other environmental stressors.

To enhance the resilience of kelp aquaculture, we propose a multifaceted mitigation strategy: first, advancing research on stress physiology to identify tolerance thresholds and acclimation mechanisms under combined stressors; second, deploying real-time monitoring systems, such as IoT-enabled sensors and machine learning-based forecasting models, to improve early warning capabilities; third, implementing ecosystem-based management practices, including the restoration of coastal buffer zones and stricter nutrient load regulations; and fourth, developing adaptive cultivation techniques, such as breeding heat-resistant kelp strains and adopting modular farming systems that can adjust to changing conditions. Additionally, our findings highlight the potential of precision fertilization within zoned management areas, where optimized timing, dosage, and application methods can effectively address nutrient deficiencies without exacerbating eutrophication risks. By integrating these approaches, the kelp aquaculture sector can better navigate the challenges posed by climate change and environmental degradation, ensuring its sustainability as a critical component of the future blue economy.

## 4. Materials and Methods

### 4.1. Algal Collection and Maintenance

Forty sporophytes of *S. japonica* were collected from the farming area in Rongcheng, Shandong Province, Northern China (37°07′ N, 120°19′ E), in December 2022. The sporophytes were approximately 60 to 70 cm in length. These sporophytes were promptly transported to the laboratory at low temperatures in insulated cooler boxes. The sporophytes were selected and rinsed several times with sterilized seawater to remove epiphytic organisms and detritus. Disks (1 cm in diameter) were then collected from the meristematic tissue zone (15 cm from the blade–stipe junction) of each sporophyte. They were then cultured in several 8 L plastic tanks containing 6 L of sterilized seawater to a density of 4 g L^−1^ at 12 °C, with an irradiance of 90 μmol photons m^−2^ s^−1^, under a 12:12 h light/dark cycle, for 3 days, for subsequent experiments. The sterilized seawater was renewed every day.

### 4.2. Culture Experiment

A culture experiment was carried out for 6 days under three N:P ratio levels (10:1, 100:1, and 500:1) combined with two temperature and light intensity conditions (12 °C, 90 μmol photons m^−2^ s^−1^, normal temperature and light intensity; 17 °C, 340 μmol photons m^−2^ s^−1^, high temperature and light intensity). One control group and three treatment groups were implemented, each with three replicates. The control group was set at a N:P ratio of 10:1 (N: 1 mg L^−1^, P: 0.1 mg L^−1^) under the normal-temperature and -light conditions. The treatment groups were as follows: the T1 group had a N:P ratio of 100:1 (N: 1 mg L^−1^, P: 0.01 mg L^−1^, phosphorus deficiency) under the normal-temperature and -light conditions, the T2 group had a N:P ratio of 100:1 (N: 1 mg L^−1^, P: 0.01 mg L^−1^, phosphorus deficiency) under the high-temperature and -light conditions, and the T3 group had a N:P ratio of 500:1 (N: 1 mg L^−1^, P: 0.002 mg L^−1^, extreme phosphorus deficiency) under the high-temperature and -light conditions. The same nitrogen and different phosphorus concentrations in the culture media were prepared by adding sodium nitrate and potassium dihydrogen phosphate (AR, Tianjin Hengxing Chemical Reagent Manufacturing Co., Ltd., Tianjin, China) to sterile filtered seawater. Well-grown disks were randomly selected and added to an 8 L glass tank containing 6 L of prepared medium, maintaining an initial density of 1.5 g L^−1^. These disks were maintained in an intelligent illumination incubator (GXZ–500, Ningbo Jiangnan Instrument Factory, Ningbo, China) under a 12:12 h light/dark cycle and the specified temperature and light conditions mentioned above. The culture medium was renewed every 3 days.

### 4.3. Ultrastructural Observation

The ultrastructural changes in algal cells from each group were observed using a transmission electron microscope (TEM; JEM–1200EX, Tokyo, Japan) after the culture experiment. Algal samples from each group were randomly selected and cut into small pieces (1 mm^3^). The samples were immediately transferred to 0.1 mol L^−1^ glutaraldehyde fixative solution prepared in phosphate buffer (pH 7.2) at 4 °C in the dark for 24 h. After being rinsed thrice with 0.1 mol L^−1^ phosphate buffer, the samples were further fixed with 1% osmium tetroxide and then dehydrated in a graded ethanol series (50%, 70%, 90%, and 100%). The fixed samples were embedded in Spurr’s resin and prepared into ultrathin sections of 50 nm in thickness by an ultramicrotome (Ultracut E, Reichert–Jung, Vienna, Austria). The ultrathin samples were stained with uranyl acetate and lead citrate for observation.

### 4.4. Measurements of the Relative Growth Rate

The fresh weights of all disks before and after the culture experiment were measured. The relative growth rate (RGR; % day^−1^) of each replicate was calculated using the following equation:

RGR (% day^−1^) = (lnW_t_ − ln W_0_)/t × 100% (1)

where W_0_ is the initial fresh weight, W_t_ is the final fresh weight, and t is the time of culture in days. After the 6-day culture, 5 algal disks were randomly taken from the control group and each of the 3 treatment groups to record the morphological status by photograph.

### 4.5. Measurements of Pigment Content

An approximately 0.1 g fresh-weight algal sample was used for Chl *a* and Chl *c* extraction in each replicate. Each sample was ground with 4 mL of 90% acetone under ice bath conditions and then placed at 4 °C in the dark for 24 h. Extracts were diluted to 10 mL and allowed to stand for 10 min. Then, these extracts were centrifuged for 20 min at 4000 rpm at 4 °C. The supernatant was collected, and the absorption was measured at 630, 645, 663, and 750 nm. The Chl *a* and Chl *c* content (mg g^−1^) was calculated using the following equations:

Chl *a* (mg g^−1^) = [11.64 × (A_663_ − A_750_) − 2.16 × (A_645_ − A_750_) + 0.10 × (A_630_ − A_750_)] × Ve/(I × W × 1000) (2)

Chl *c* (mg g^−1^) = [54.22 × (A_630_ − A_750_) − 14.18 × (A_645_ − A_750_) − 5.53 × (A_663_ − A_750_)] × Ve/(I × W × 1000) (3)

where A_750–625_ is the absorption of extracts at 630, 645, 663, and 750 nm; Ve is the volume of DMF (mL); I is the optical path in the cuvette (cm); and W is the fresh weight of the samples (g).

Fucoxanthin extraction was conducted using approximately 0.02 g of lyophilized algal powder per replicate. Fresh algal biomass was initially freeze-dried (FD-1A-50, Boyikang Laboratory Instruments Co., Beijing, China) and mechanically homogenized. The processed material was fractionated through a 60-mesh sieve to obtain standardized particulates. Accurately weighed aliquots of the desiccated powder were transferred to aluminum-foil-encased 10 mL centrifuge tubes to prevent photodegradation. A dual-phase extraction solvent (90% ethanol/acetone, 3:1 *v*/*v*) was introduced at a mass-to-volume ratio of 1:40 (*w*/*v*). Sequential thermal extractions were conducted in a precision-controlled water bath (65 °C) with dual 80 min incubation intervals. Following phase separation, the mixtures were centrifuged at 5000 rpm for 10 min (4 °C) to isolate the supernatants. The extracted fractions were analyzed for absorbance at 450 nm. The fucoxanthin content was calculated according to the following formula:

Fucoxanthin (mg g^−1^) = 1000 × A_450_ × V/(1600 × M × 100) (4)

where A_450_ is the absorbance of extracts at 450 nm, V is the total extraction volume (mL), and M is the mass of dried algal powder (g).

### 4.6. Measurements of Chlorophyll Fluorescence Parameters

The maximum photochemical quantum yield (F_v_/F_m_), the effective quantum yield of PSII (ΦPSII), and the non-photochemical quenching (NPQ) of the algal sample from each replicate were estimated using a pulse amplitude modulated fluorometer (Dual–PAM–100, Walz, Effecltrich, Germany) after 24 h of incubation. Before measurement, the algal samples were placed in the dark for 20 min. F_v_/F_m_, ΦPSII, and NPQ were calculated with the following formulas:

F_v_/F_m_ = (F_m_ − F_0_)/F_m_ (5)

ΦPSII = (F_m_′ − F_s_)/F_m_′ (6)

NPQ = (F_m_ − F_m_′)/F_m_′ (7)

where F_0_ and F are the steady-state fluorescence measured in the dark and light, respectively, and F_m_ and F_m_′ are the maximum fluorescence measured after exposure to a saturation pulse in the dark and light, respectively.

### 4.7. Measurements of AKP and ACP Activities

A 0.1 g fresh-weight algal sample was used to measure the activity of alkaline phosphatase (AKP) and acid phosphatase (ACP) in each replicate. The samples were immediately frozen with liquid nitrogen and stored at −80 °C until analysis. The AKP and ACP activities were measured using an alkaline phosphatase assay kit (Nanjing Jiancheng Bioengineering Institute, Nanjing, China) and an acid phosphatase assay kit (Nanjing Jiancheng Bioengineering Institute, Nanjing, China), respectively. All experimental procedures strictly adhered to the manufacturer’s instructions.

### 4.8. Metabolite Extraction

Approximately 0.1 g of the samples from each replicate were separately homogenized using liquid nitrogen. Pre-chilled 80% methanol and 0.1% formic acid were used for metabolite extraction. The mixture of the sample and extraction solution was centrifuged at 15,000× *g* and 4 °C for 5 min. Supernatants were diluted with LC/MS-grade water until the methanol concentration inside reached 53%. Diluted supernatants were conveyed to new tubes and centrifuged at 15,000× *g* and 4 °C for 20 min. The final supernatants were collected for analysis and quality control. Then, the supernatants were analyzed using untargeted metabolome analysis through a UHPLC system and a mass spectrometer (Thermo Fisher Scientific, Waltham, MA, USA).

The extracts were injected into a Hyperil Gold column (100 × 2.1 mm, 1.9 μm) using a 12 min linear gradient with a flow rate of 0.2 mL min^−1^. Eluent A (0.1% formic acid) and eluent B (methanol) were utilized in the positive polarity mode. Eluent A (5 mM ammonium acetate, pH 9.0) and eluent B (methanol) were utilized in the negative polarity mode. The solvent gradient was set as follows: 0–1.5 min, 2% B; 1.5–3 min, 2–85% B; 3–10 min, 85–100% B; 10–10.1 min, 100–2% B; 10.1–11 min, 2% B; and 11–12 min, 2% B. The operating conditions of the mass spectrometer were as follows: a spray voltage of 3.5 kV, a capillary temperature of 320 °C, a sheath gas flow rate of 35 psi, an aux gas flow rate of 10 L min^−1^, an S-lens RF level of 60, and an aux gas heater temperature of 350 °C.

### 4.9. Data Analysis

A one-way ANOVA was employed to explore the RGR, Chl *a* content and chlorophyll fluorescence parameters (F_v_/F_m_, ΦPSII, and NPQ) of *S. japonica* sporophytes among different groups. A two-way ANOVA was used to analyze the activities of AKP and ACP among different groups. Before the analyses, the normality of the data and the homogeneity of variance were checked. Once the significance of the differences (*p* < 0.05) was tested, Tukey’s multiple comparison test was performed. All processes mentioned above were completed through the SPSS statistical software (version 26.0).

Through the UHPLC–MS/MS analyses, metabolomic raw data were obtained and then selected for peak alignment and picking, as well as metabolite quantitation. Metabolites were annotated using the KEGG database. PCA was analyzed using the R package ropls (Version 1.6.2). The differential metabolites (DMs) between the treatment groups and the control group were identified using the following criteria: variable importance in projection (VIP) > 1, *p* < 0.05, and fold change (FC) ≥ 1.5. Differential metabolites among the two groups were mapped into their biochemical pathways through metabolic enrichment and pathway analysis based on the KEGG database (http://www.genome.jp/kegg/ accessed on 15 July 2024).

## 5. Conclusions

Our results provide compelling evidence supporting the hypothesis that phosphorus limitation constituted the primary stressor, whose synergistic interaction with thermal and high-light stress likely precipitated the 2021 kelp aquaculture collapse in Rongcheng. This study reveals that *S. japonica* employs a complex adaptive strategy wherein phosphorus scarcity initiates metabolic conservation mechanisms; concurrent thermal/light stress overrides these phosphorus-limited conditions by activating specific compensatory pathways; and metabolic trade-offs mediate acclimation to these multiple stressors. This sophisticated regulatory system underscores *S. japonica*’s capacity to dynamically balance nutrient limitation and environmental stress through remarkable metabolic plasticity. These mechanistic insights establish a scientific foundation for developing stress-resilient kelp varieties, optimizing aquaculture management practices, and enhancing sustainable kelp production under climate change scenarios.

## Data Availability

The data will be made available on request.
